# Family social support during incarceration: implications for health upon release

**DOI:** 10.1038/s41598-025-11274-6

**Published:** 2025-07-29

**Authors:** Chantal Fahmy, Alexander Testa

**Affiliations:** 1 Department of Criminology and Criminal Justice, The University of Texas at San Antonio, 501 West Cesar E. Chavez Blvd., San Antonio, TX 78207 USA; 2 Department of Management, Policy, and Community Health, University of Texas Health Science Center at Houston, Houston, USA

**Keywords:** Public health, Risk factors

## Abstract

Incarceration is associated with adverse physical and mental health that are often brought to light during reentry into the community, particularly in the immediate period following release. Social support, specifically from family members, has been identified as a key protective factor that may promote health and reintegration success among formerly incarcerated individuals. However, less is known about how specific types of family support—emotional and instrumental—relate to health outcomes following release from incarceration. The current study uses data from 517 individuals incarcerated in a large Texas prison, surveyed before and approximately one month after release, to examine the relationship between family support and self-rated physical and mental health. Logistic regression models revealed that strong emotional family support was significantly associated with better self-rated physical health and mental health one month post-release. Additionally, strong instrumental family support predicted better mental health but not physical health among respondents. These findings highlight the crucial role of emotional and instrumental familial support systems in fostering and reducing health disparities and promoting equity among justice-impacted populations.

**Supplementary Information:**

The online version contains supplementary material available at 10.1038/s41598-025-11274-6.

## Introduction

With nearly 1.8 million individuals incarcerated on any given day^1^, the United States (U.S.) has an incarceration rate that stands approximately 7 to 10 times greater than that of other developed democracies^2,3^. The vast size and scope of incarceration has placed the U.S. in an era of mass incarceration for over five decades^4^, which has generated far-reaching consequences, not only for those who experience confinement but also for their families^5^, communities^6^, and broader societal structures^7,8^. Indeed, for individuals with lived experiences of serving time incarcerated the adverse effects of incarceration extend well beyond the prison walls, disrupting social networks^9–11^, perpetuating economic instability^12^, and exacerbating health disparities^2,13,14^.

The health implications of incarceration and reentry are particularly alarming, as incarceration is consistently linked to profound and lasting physical and mental health challenges, which are often exacerbated after release and during reintegration into the community^13–15^. A substantial body of research demonstrates that incarcerated individuals experience disproportionately high rates of chronic illness, infectious disease, and mental health disorders compared to the general population^16^, with limited access to adequate healthcare while confined^17–19^. These health disparities do not end upon release; rather, they often intensify as individuals face significant barriers to healthcare access, heightened stressors related to reintegration, and cumulative effects of social and economic instability^15,20–24^. The reentry period is a particularly vulnerable time, marked by increased risks of untreated medical conditions^25–29^, relapse into substance use^30,31^, and elevated mortality rates^32^. Given the structural and social challenges that formerly incarcerated individuals encounter, addressing the enduring health consequences of incarceration is critical for promoting successful reintegration and reducing health disparities in justice-impacted populations.

Given these challenges, identifying protective factors that promote successful reentry and mitigate the negative health effects of incarceration is critical^33,34^. One of the most significant protective factors in this process is social support—particularly support from family members^35–39^. Family members often serve as the primary source of support, providing both emotional and instrumental resources that can enhance reintegration success^40^. Emotional support, such as encouragement, reassurance, and understanding, can help individuals cope with the psychological distress associated with reentry and build a foundation necessary for overall health^41,42^. Instrumental support, including assistance with securing housing, employment, and transportation, directly addresses practical barriers to stability and self-sufficiency, thus reducing stressors and hardships that undermine wellbeing^41,43^.

Despite the recognized importance of family support, particularly as an untapped resource, there remains a need for empirical research examining how different types of familial support influence health outcomes among formerly incarcerated individuals. Indeed, Mowen et al. (2019) sought to move beyond “if” family support matters and delve into “why” it matters. Their aim was to understand if both emotional support and instrumental support perspectives from family members carry explanatory power during reentry. They found that family instrumental support was associated with lower levels of substance use and criminal offending and lower odds of reincarceration, but not emotional support. Similarly, Liu et al.^44^ examined multiple sources of social support (e.g., family, religious, parole officer) and that the role of general family support was still salient, despite when other forms of social support were included. Both studies focused their outcomes of interest on recidivism and substance use during reentry, which, albeit important in the study of incarceration and reintegration, may not paint the whole picture of the indirect yet significant factors, such as health, of consequence in the reentry journey. Although Fahmy^40^ explored outcomes of physical and mental health, their focus was on the *stability* of social support across various domains and extended during the first year post-release, instead of shortly after release.

Thus, the current study seeks to fill this gap in the literature by investigating the relationships between family emotional and instrumental support measured prior to prison release and self-rated physical and mental health measured in approximately one month after release from prison. Using data collected from 517 individuals prior to and following release from a large prison facility in Texas, we test the following hypotheses:

- **H1:** Greater family emotional support prior to release from prison will be associated with better self-rated physical health one month following release from prison.
- **H2:** Greater family emotional support prior to release from prison will be associated with better self-rated mental health one month following release from prison.
- **H3:** Greater family instrumental support prior to release from prison will be associated with better self-rated physical health one month following release from prison.
- **H4:** Greater family instrumental support prior to release from prison will be associated with better self-rated mental health one month following release from prison.

## Methods

### Data

This study uses data from the LoneStar Project—a longitudinal study of men released from two prisons in Texas, the largest state correctional department, housing the most incarcerated people per state^45^. Approximately 95% of baseline interviews were conducted in the Huntsville Unit, which is Texas’s largest release center^46^ and the remaining 5% of baseline interviews were conducted in the nearby Estelle Unit. The initial stages of the study consisted of intensive interviews with respondents (*N* = 802) in prison approximately 1 week prior to release (baseline;^47^) and again approximately 1 month later (wave 2) in the community over the phone (*n* = 532;^48^). The first month of post-incarceration is a critical time period with respect to health and community reintegration outcomes^24,40^. The sample composition is consistent with prison releasees from the Texas Department of Criminal Justice (TDCJ) with a mean age of 39, a larger than average Latino/Hispanic population compared to other states (approximately 39%), a majority with a high school education or GED (45%), 70% with at least one minor child, a large proportion of those with gang affiliation (46%), and most having served time incarcerated at least once in a TDCJ facility. All methods were carried out in accordance with relevant guidelines and regulations and were approved by the Arizona State University Institutional Review Board. Moreover, informed consent was obtained from all participants.

### Dependent variables

The dependent variables are self-rated *physical health* and *mental health* at wave 2. Respondents were asked, “Would you say your general physical health is excellent, good, fair, or poor?” and were asked the same question regarding mental health. In both cases, fair and poor health were collapsed into one category (= 0) and good/excellent health were collapsed into one category (= 1), creating binary variables to ensure adequate cell sizes for analyses. Thus, scores of 1 reflect better health for both dependent variables. About 78% of respondents rated their physical health as good or excellent and similarly, approximately 77% of respondents rated their mental/emotional health as good or excellent. The original distribution of the health variables prior to transformation are provided in Appendix A.

### Independent variables

The final independent variables, both measured at the baseline interview in prison, are a series of three dichotomous items stemming from ordinal level measures of *family emotional social support* and *family instrumental social support*. These measures were derived from the Multidimensional Scale of Perceived Social Support (MSPSS) which was initially developed and validated using subscales for family, friends, and significant others^49^. The original emotional support variables were comprised of three statements reflecting level of agreement including, “You have someone in your family who…” “is willing to help you make decisions”, “really tries to help you”, and “can give you the emotional help and support you need”. The original instrumental support variables were comprised of five statements reflecting level of agreement including, “You have someone in your family who would provide…” “help or advice on finding a place to live”, “help or advice on finding a job”, “support for dealing with a substance abuse problem” “transportation to work or other appointments if needed” and “financial support”. A couple steps were conducted to attain the final variables used in the models: low social support (the reference in all four models), moderate social support, and high social support. First, the three emotional support statements and the five instrumental support statements were combined into two overall measures representing total emotional support and total instrumental support; each of which were coded as 0 for “strongly disagree”, 1 for “disagree”, 2 for “agree” and 3 for “strongly agree” per statement. Second, with a range of 0–9 for emotional support (summation of three items) and 0–15 for instrumental support (summation of five items), the variables were recoded to represent *low social support* (comprised of scores of 0–3 for emotional support and 0–5 for instrumental support), *moderate social support* (comprised of scores of 4–6 for emotional support and 6–10 for instrumental support), and *high social support* (comprised of scores of 7–9 for emotional support and 11–15 for instrumental support). The cut points used for low, moderate, and high levels of social support were to ensure roughly equivalent sized bins across levels of agreement. Table 1 displays the level of support across the six binary measures. About 8% of respondents had low emotional support and nearly 9% reported low instrumental support, 26% had moderate emotional support and about 27% had moderate instrumental support, and nearly 65% had high emotional support while almost 62% had high instrumental support from family. Please see Appendix A for the original distribution of the family social support statements.

**Table 1:** Descriptive statistics (*N* = 517).

Variables	Mean or %	Standard deviation	Minimum	Maximum
Dependent Variables (Wave 2)				
Physical health (good/excellent)	78.5%	–	0	1
Mental health (good/excellent)	77.4%	–	0	1
Independent Variables (Baseline)				
Family emotional social support				
Low social support (*reference*)	8.1%	–	0	1
Moderate social support	26.0%	–	0	1
High social support	64.9%	–	0	1
Family instrumental social support				
Low social support (*reference*)	8.7%	–	0	1
Moderate social support	26.6%	–	0	1
High social support	61.7%	–	0	1
Control Variables				
Respondent age	39.06	11.22	19	73
White (*reference*)	28.9%	–	0	1
Black	26.0%	–	0	1
Latino/Hispanic	39.3%	–	0	1
Other race	5.9%	–	0	1
Eighth grade or less	14.6%	–	0	1
Some high school (*reference*)	44.8%	–	0	1
High school graduate	25.8%	–	0	1
College	14.7%	–	0	1
Married	24.9%	–	0	1
Has children	70.1%	–	0	1
Chronic disease	18.2%	–	0	1
TDCJ gang status	45.9%	–	0	1
Number of times previously incarcerated	1.99	1.24	1	9
Incarceration length (years)	6.23	6.32	0	36
Violent incarcerating offense	16.8%	–	0	1
Property incarcerating offense (*reference*)	19.7%	–	0	1
Drug incarcerating offense	39.5%	–	0	1
Other incarcerating offense	23.9%	–	0	1

Physical health and mental health are both self-rated measures.

*TDCJ =* Texas Department of Criminal Justice.

### Control variables

Consistent with prior research, we controlled for a range of measures commonly associated with incarceration, social support, and health. The covariates are related to a respondent’s demographics, health status, and incarceration history. Demographic measures include *respondent age* in years; race/ethnicity (*White* as the reference category, *Black*, *Latino/Hispanic*, and *other race*); education (*eighth grade or less*, *some high school* [reference], *high school graduate*, and *college*; if the respondent is *married* (1 = yes, 0 = no); or *has children* (1 = yes, 0 = no); and if the respondent replied “yes” (= 1) to having a chronic disease which includes HIV/AIDS, hepatitis B or hepatitis C, anemia, or a seizure disorder during the baseline interview. Incarceration-related covariates include the respondent’s *TDCJ gang status* (46% said “yes”); a continuous level variable on the *number of times previously incarcerated* in a TDCJ prison (M = 2 stints), the current *incarceration length* in years (M = 6.23 years), and a series of binary variables indicating the current incarcerating offense: *violent* (17%), *property* (20%, reference), *drug* (39%), and *other* (24%). See Table 1 for details on the descriptive statistics of all sample variables.

### Analytic strategy

The analytic strategy proceeds in two steps. First, prior to running all multivariable models, correlations, variance inflation factors (VIFs), and tolerance levels of collinearity and heteroskedasticity among key variables are assessed—all of which fall within disciplinary standards. Specifically, the mean VIF across all study variables was 3.27, demonstrating that multicollinearity was not a concern^50^. Second, logistic regression models are employed to assess the association between family emotional and instrumental support on physical and mental health. Model 1 in Table 2 examines physical health regressed on family emotional social support while Model 2 in Table 2 examines mental health regressed on family emotional social support. Similarly, Model 3 in Table 3 examines physical health regressed on family instrumental social support, while Model 4 in Table 3 examines mental health regressed on family instrumental social support. Details of this second step in the analytic strategy can be found in Tables 2 and 3. All four logistic regression models display odds ratios (OR) with 95% robust confidence intervals (CI) and all multivariable models are estimated using Stata version 18.5 (StataCorp).

**Table 2:** Logistic regression models of emotional support predicting health at wave 2 (*N* = 517).

Variables	Physical Health		Mental Health	
	Model 1		Model 2	
	OR	95% CI	OR	95% CI
Family Emotional Social Support				
Low social support (*reference*)	–	–	–	–
Moderate social support	2.316	(0.941–5.700)	1.128	(0.479–2.653)
High social support	3.483**	(1.488–8.150)	2.367*	(1.039–5.396)
Control Variables				
Respondent age	0.952**	(0.926–0.981)	0.975	(0.950–1.001)
White (*reference*)	–	–	–	–
Black	0.627	(0.317–1.240)	0.389**	(0.206–0.737)
Latino/Hispanic	0.463*	(0.237–0.906)	0.846	(0.452–1.586)
Other race	0.459	(0.144–1.463)	0.822	(0.251–2.690)
Eighth grade or less	1.001	(0.484–2.075)	0.790	(0.408–1.530)
Some high school (*reference*)	–	–	–	–
High school graduate	0.967	(0.528–1.769)	1.097	(0.587–2.049)
College	1.393	(0.712–2.724)	1.428	(0.753–2.709)
Married	1.020	(0.580–1.708)	1.923*	(1.099–3.362)
Has children	0.945	(0.558–1.602)	0.822	(0.499–1.354)
Chronic disease	0.431**	(0.246–0.753)	0.397**	(0.236–0.677)
TDCJ gang status	1.556	(0.893–2.712)	1.244	(0.763–2.028)
Number of times previously incarcerated	0.875	(0.718–1.067)	0.873	(0.720–1.059)
Incarceration length (years)	1.026	(0.984–1.067)	0.997	(0.958–1.037)
Violent incarcerating offense	1.604	(0.817–3.150)	1.563	(0.761–3.210)
Property incarcerating offense (*reference*)	–	–	–	–
Drug incarcerating offense	2.650**	(1.393–5.043)	1.417	(0.762–2.635)
Other incarcerating offense	3.251**	(1.546–6.834)	1.379	(0.670–2.837)
*R*^2^	0.152		0.119	

Entries are odds ratios (OR) and 95% robust confidence intervals (CI).

***p < 0.001, **p < 0.01, *p < 0.05.

**Table 3:** Logistic regression models of instrumental support predicting health at wave 2 (*N* = 517).

Variables	Physical Health		Mental Health	
	Model 3		Model 4	
	OR	95% CI	OR	95% CI
Family Instrumental Social Support				
Low social support (*reference*)	–	–	–	–
Moderate social support	1.421	(0.578–3.493)	1.319	(0.576–3.023)
High social support	2.246	(0.932–5.413)	2.483*	(1.099–5.609)
Control Variables				
Respondent age	0.954**	(0.928–0.982)	0.979	(0.954–1.005)
White (*reference*)	–	–	–	–
Black	0.659	(0.332–1.310)	0.374**	(0.193–0.722)
Latino/Hispanic	0.478*	(0.243–0.941)	0.814	(0.425–1.561)
Other race	0.382	(0.122–1.189)	0.614	(0.198–1.902)
Eighth grade or less	0.919	(0.441–1.914)	0.741	(0.385–1.423)
Some high school (*reference*)	–	–	–	–
High school graduate	1.022	(0.569–1.835)	1.160	(0.635–2.120)
College	1.398	(0.727–2.689)	1.413	(0.746–2.677)
Married	1.054	(0.615–1.809)	1.883*	(1.086–3.266)
Has children	0.915	(0.543–1.543)	0.827	(0.504–1.358)
Chronic disease	0.435**	(0.251—0.754)	0.397***	(0.237—0.666)
TDCJ gang status	1.547	(0.887–2.700)	1.214	(0.746–1.975)
Number of times previously incarcerated	0.870	(0.712–1.063)	0.868	(0.716–1.053)
Incarceration length (years)	1.025	(0.983–1.068)	0.993	(0.954–1.033)
Violent incarcerating offense	1.692	(0.860–3.327)	1.627	(0.789–3.351)
Property incarcerating offense (*reference*)	–	–	–	–
Drug incarcerating offense	2.678**	(1.402–5.116)	1.477	(0.792–2.755)
Other incarcerating offense	2.996**	(1.456–6.166)	1.347	(0.667–2.719)
*R*^2^	0.139		0.105	

Entries are odds ratios (OR) and 95% robust confidence intervals (CI).

***p < 0.001, **p < 0.01, *p < 0.05.

## Results

Table 1 presents the descriptive statistics of the analytic sample with a final sample size of 517. The analysis was restricted to those who participated in both the baseline in-prison interview and wave 2 post-release interview as well as who were not missing on key variables such as physical and mental health. Using the same dataset, Mitchell et al.^51^ found that listwise deletion of missing cases across waves produced the most similar results to that of the full sample compared to other approaches to missingness such as multiple imputation. See Clark et al.^52^ for an in-depth analysis of attrition in the LoneStar Project. Most of the sample reported good or excellent physical and mental health (77–79%), a result that is contrary to many expectations of formerly incarcerated persons’ health, but has nevertheless been recorded in previous research^20,53^. Similarly, during the baseline interview, a majority of LoneStar project participants agreed or strongly agreed with positive statements conceptualizing emotional familial support (between 87 and 89%) and instrumental familial support (between 80 and 90%; see Appendix A for details). Thus, translating to higher levels of moderate and high family emotional support (nearly 96%) as well as moderate and high family instrumental support (about 88%).

Table 2 presents the results from the multivariable logistic regression models of family emotional social support predicting physical health (Model 1) and mental health (Model 2) at wave 2. Both models had significant Wald chi-square test statistics (Model 1 χ^2^ = 67.32, *p* < 0.001; Model 2 χ^2^ = 50.72, *p* < 0.001), indicating good model fit^54^. Across both models, low social support is set as the reference category. Turning to Model 1 in Table 2, moderate family emotional social support is not associated with physical health (although the* p*-value increases to 0.068), while high family emotional social support shows a strong association with physical health (odds ratio [OR] 3.48; 95% Confidence Interval [CI] [1.49, 8.15]). In Model 2 only high emotional social support, not moderate support, from family is associated with positive mental health (OR 2.37; CI [1.04, 5.40]). Respondent age and having a chronic disease are negatively associated with positive physical health, whereas either being incarcerated for a drug or other offense are positively associated with physical health. In terms of the covariates in the emotional support and mental health model, being married is positively associated with mental health whereas identifying as Black and again, having a chronic disease are both negatively associated with self-reported mental health.

Table 3 presents the results from the multivariable logistic regression models of family instrumental social support predicting physical health (Model 3) and mental health (Model 4) at wave 2. As with Table 2, both Models 3 and 4 have significant Wald chi-square test statistics (Model 3 χ^2^ = 59.82, *p* < 0.001; Model 4 χ^2^ = 46.81, *p* < 0.002). In both models in Table 3, low social support is the reference category. Turning to Model 3 in Table 3, neither of the family instrumental support levels are associated with physical health post-release. However, similar to both Models 1 and 2 in Table 2, Model 4 also shows a significant positive association but only between high social support and self-rated mental health (OR = 2.48; CI = [1.10, 5.61]); not simply a moderate level of instrumental support. In terms of covariates associated with both physical and mental health in Table 3, the same variables are significant in the same directions as in Table 2. For physical health, that is respondent age, chronic disease, a drug incarcerating offense, and an other incarcerating offense, and for mental health, it is being married, identifying as Black, and having a chronic disease.

## Discussion

The first month after release from incarceration is fraught with a plethora of challenges and expectations for the reentering person, not the least of which is considering their own health status^15,22^. Whether increased issues with one’s physical and mental health begin prior to incarceration or stem from issues heightened during incarceration^55,56^, the reentry journey is wrought by countless challenges^43,57^. One of the most important factors to support successful community reintegration, “perhaps more so than any other factor, family support has been highlighted as an extraordinarily vital component for reentry success”^37^.

The current study aimed to test the impact of family emotional support and family instrumental support on self-perceptions of physical and mental health shortly after prison release. Three of the four hypotheses—that greater social support from family reported in the days prior to release would be associated with better self-rated physical and mental health during the integral first few weeks post-release—were supported. Only in the case of Model 3, which examined levels of instrumental social support on self-assessments of physical health was Hypothesis 3 not supported. In practice this makes sense as instrumental support concerns practical assistance such as transportation or direct financial support, of which physical health may only be indirectly related. Curiously, however, in none of the models with statistically significant associations of social support and health (i.e., Models 1, 2, and 4) were both moderate and high levels of social support of consequence. In other words, there seems to be a “tipping point” in which a baseline standard of low or moderate feelings of support from family members, do not suffice. However, to this point, the odds ratios for the moderate support variables are relatively sizable, so the wide standard errors in those models should be a point of investigation in a different sample with different support variables in future research. Considering that the reentry period is highly stressful^24^, only very strong support—what ‘strongly agree’ on the original statements likely captures—has a meaningful impact. Simply marking ‘agree’ might only reflect general or occasional support from a variety of family members and thus may be insufficient to overcome structural barriers and psychological stressors upon release. For instance, prior qualitative research among formerly incarcerated adults highlights the large degree that individuals often must tap into familial support networks to overcome challenges endured in the early days following release from incarceration^41,43,58^.

Before discussing the implications of the study, there are several limitations and directions for future work that flow from the study results. First, the study relies on self-report measures of mental and physical health. Future work may aim to replicate the results using alternative measures such as clinical assessments of health and validated diagnostics of mental wellbeing. Second, the focus of the study is on the first month following release from incarceration. Accordingly, the results cannot speak to how health and social supports look after a longer duration. For instance, initial support may wane, or conversely, some individuals may build stronger support networks in the months following release. Longitudinal studies with multiple follow-ups would help capture these trajectories. Third, although our data include several important covariates, unobserved confounding remains a possibility. For example, individuals with strong family support may also possess other unmeasured assets—such as higher motivation or pre-existing community connections—that contribute to improved health outcomes. Finally, the study sample includes only individuals released from Texas prisons, which may limit generalizability to other state systems or local jail populations.

While these findings add to the extant literature on how social support impacts the health and wellbeing of formerly incarcerated individuals, the findings also point to areas in need of further research. For instance, future work can employ qualitative methods to more deeply understand the nuance of when, how, and for whom social support from family matters most for health outcomes, as well as the importance of the perceived strength of the bond^36,44,59^. Likewise, future research should explore and develop alternative measures that can tap into greater granularity of the strength and dosage of support received and accepted.

On a concluding note, the findings also have implications for criminal justice and public health policy and programs. For instance, the results suggest that correctional systems and reentry programs should prioritize family engagement and ensure social and instrumental support systems are in place prior to release from incarceration. This could include facilitating structured family preparation sessions, offering family-inclusive reentry planning, and reducing barriers to maintaining communication while incarcerated (e.g., reducing the costs of phone calls or offering virtual visitation^60–62^. Furthermore, future research should also take an in-depth look at family dynamics more broadly^63^. In time, weariness about financial struggles, overall family functioning, and increased tension may start to form^64–66^. Nevertheless, families are well positioned to aid their loved ones during this stressful period. For instance, some estimates suggest that upwards of 92% of formerly incarcerated individuals receive direct cash assistance from family members, which is crucial in the early stages of reintegration, particularly since the majority of reentrants have legal financial obligations upon release^66^. Similarly, the Returning Citizen Stimulus (RCS) program found success via significant reductions in parole violations by providing formerly incarcerated people direct cash payments in the 60 days following release for reaching personal milestones, such as completing a resume^67^.

The public and criminal justice system would benefit overall from making this point clear to families early on (i.e., from the first day of incarceration, rather than the last day); especially for those who live in rural areas or do not have suitable and accessible community support networks. On a related note, in some cases, family relationships are strained and negative emotions may be more accurate of the experiences felt by both the formerly incarcerated person and their loved ones^35,36,65^. In these situations, it may be more beneficial for all parties if the reentrant sought out community support rather than relying on their families for either emotional or instrumental support, certainly in times of family financial strain.

From a broader public health perspective, our findings point to the need for a more integrated, family-centered approach to health equity for justice-involved populations. Health systems and social service agencies can partner with reentry programs to deliver wraparound support that acknowledges the role of family in shaping post-release health. Policies should also be designed to reduce structural barriers that make it difficult for families to provide care—such as housing restrictions, income instability, and lack of paid leave—so that their support can be leveraged as a genuine protective factor^68,69^.

## Electronic supplementary material

Below is the link to the electronic supplementary material.

Supplementary Material 1

## Data Availability

Data for the current study are publicly available and can be found on the ICPSR website at https://www.icpsr.umich.edu/web/NACJD/studies/37859.
