# Supplementary material for: Family social support during incarceration: implications for health upon release
Source: Sci Rep. 2025 Jul 29;15:27716. doi: 10.1038/s41598-025-11274-6 (PMC12307891; doi:10.1038/s41598-025-11274-6)
Supplement: Supplementary file 1 — Supplementary Material 1 [file 41598_2025_11274_MOESM1_ESM.docx]

**Appendix A**

**Original Distributions of Key Variables**

**Dependent Variables (wave 2)**

| **Physical Health** | ***N*** | **Percent** |
| --- | --- | --- |
| Poor | 30 | 5.70% |
| Fair | 83 | 15.78% |
| Good | 196 | 37.26% |
| Excellent | 217 | 41.25% |

| **Mental Health** | ***N*** | **Percent** |
| --- | --- | --- |
| Poor | 25 | 4.74% |
| Fair | 94 | 17.84% |
| Good | 190 | 36.05% |
| Excellent | 218 | 41.37% |

**Independent Variables (baseline)**

**Family Emotional Social Support Statements**

*You have someone in your family who…*

| 1. **Is willing to help you make decisions** | ***N*** | **Percent** |
| --- | --- | --- |
| Strongly Disagree | 42 | 5.27% |
| Disagree | 57 | 12.42% |
| Agree | 273 | 34.25% |
| Strongly Agree | 425 | 53.32% |

| 1. **Really tries to help you** | ***N*** | **Percent** |
| --- | --- | --- |
| Strongly Disagree | 30 | 3.76% |
| Disagree | 48 | 6.02% |
| Agree | 221 | 27.73% |
| Strongly Agree | 498 | 62.48% |

| 1. **Can give you the emotional help**   **and support you need** | ***N*** | **Percent** |
| --- | --- | --- |
| Strongly Disagree | 34 | 4.28% |
| Disagree | 55 | 6.93% |
| Agree | 259 | 32.62% |
| Strongly Agree | 446 | 56.17% |

**Family Instrumental Social Support Statements**

*You have someone in your family who would provide…*

| 1. **Help or advice on finding a place to live** | ***N*** | **Percent** |
| --- | --- | --- |
| Strongly Disagree | 27 | 3.39% |
| Disagree | 50 | 6.27% |
| Agree | 268 | 33.63% |
| Strongly Agree | 452 | 56.71% |

| 1. **Help or advice on finding a job** | ***N*** | **Percent** |
| --- | --- | --- |
| Strongly Disagree | 29 | 3.64% |
| Disagree | 65 | 8.16% |
| Agree | 277 | 34.76% |
| Strongly Agree | 426 | 53.45% |

| 1. **Support for dealing with a substance abuse problem if you had one** | ***N*** | **Percent** |
| --- | --- | --- |
| Strongly Disagree | 45 | 5.67% |
| Disagree | 72 | 9.07% |
| Agree | 263 | 33.12% |
| Strongly Agree | 414 | 52.14% |

| 1. **Transportation to work or other appointments if needed** | ***N*** | **Percent** |
| --- | --- | --- |
| Strongly Disagree | 36 | 4.51% |
| Disagree | 80 | 10.03% |
| Agree | 261 | 32.71% |
| Strongly Agree | 421 | 52.76% |

| 1. **Financial support** | ***N*** | **Percent** |
| --- | --- | --- |
| Strongly Disagree | 46 | 5.78% |
| Disagree | 109 | 13.69% |
| Agree | 276 | 34.67% |
| Strongly Agree | 365 | 45.85% |

**Cross-Tabulations of Emotional Social Support and Instrumental Social Support**

**Emotional Support Instrumental Support**

|  | Low social support | Moderate social support | High social support | **Total** |
| --- | --- | --- | --- | --- |
| Low social support | 48  6.07 | 13  1.64 | 3  0.38 | 64  8.09 |
| Moderate social support | 15  1.90 | 172  21.74 | 24  3.03 | 211  26.68 |
| High social support | 6  0.76 | 49  6.19 | 461  58.28 | 516  65.23 |
| **Total** | 69  8.72 | 234  29.58 | 488  61.69 | 791  100.00 |

Frequencies and cell percentages reported. Pearson χ^2^ (4) = 805.5691. Pr = 0.000

**Numbered Variable List for Correlation Matrix**

1. Physical health
2. Mental health
3. Family emotional social support
4. Family instrumental social support
5. Respondent age
6. White (*reference*)
7. Black
8. Latino/Hispanic
9. Other race
10. Eighth grade or less
11. Some high school (*reference*)
12. High school graduate
13. College
14. Married
15. Has children
16. Chronic disease
17. TDCJ gang status
18. Number of times previously incarcerated
19. Incarceration length (years)
20. Violent incarcerating offense
21. Property incarcerating offense (*reference*)
22. Drug incarcerating offense
23. Other incarcerating offense

**Bivariate Correlation Matrix of Study Variables**

|  | **1** | **2** | **3** | **4** | **5** | **6** | **7** | **8** | **9** | **10** | **11** | **12** | **13** | **14** | **15** | **16** | **17** | **18** | **19** | **20** | **21** | **22** | **23** |
| --- | --- | --- | --- | --- | --- | --- | --- | --- | --- | --- | --- | --- | --- | --- | --- | --- | --- | --- | --- | --- | --- | --- | --- |
| **1** | 1 |  |  |  |  |  |  |  |  |  |  |  |  |  |  |  |  |  |  |  |  |  |  |
| **2** | 0.43 | 1 |  |  |  |  |  |  |  |  |  |  |  |  |  |  |  |  |  |  |  |  |  |
| **3** | 0.2 | 0.18 | 1 |  |  |  |  |  |  |  |  |  |  |  |  |  |  |  |  |  |  |  |  |
| **4** | 0.16 | 0.17 | 0.79 | 1 |  |  |  |  |  |  |  |  |  |  |  |  |  |  |  |  |  |  |  |
| **5** | -0.24 | -0.16 | -0.18 | -0.19 | 1 |  |  |  |  |  |  |  |  |  |  |  |  |  |  |  |  |  |  |
| **6** | 0.01 | 0.02 | -0.12 | -0.13 | 0.15 | 1 |  |  |  |  |  |  |  |  |  |  |  |  |  |  |  |  |  |
| **7** | 0.04 | -0.1 | 0.09 | 0.09 | -0.05 | -0.38 | 1 |  |  |  |  |  |  |  |  |  |  |  |  |  |  |  |  |
| **8** | -0.02 | 0.07 | 0.07 | 0.08 | -0.1 | -0.51 | -0.48 | 1 |  |  |  |  |  |  |  |  |  |  |  |  |  |  |  |
| **9** | -0.06 | -0.01 | -0.09 | -0.06 | 0.01 | -0.16 | -0.15 | -0.2 | 1 |  |  |  |  |  |  |  |  |  |  |  |  |  |  |
| **10** | -0.01 | -0.03 | -0.03 | -0.04 | -0.07 | -0.05 | -0.1 | 0.14 | -0.01 | 1 |  |  |  |  |  |  |  |  |  |  |  |  |  |
| **11** | -0.03 | -0.05 | -0.05 | -0.01 | -0.09 | -0.11 | 0.02 | 0.09 | -0.01 | -0.37 | 1 |  |  |  |  |  |  |  |  |  |  |  |  |
| **12** | 0.03 | 0.05 | 0.11 | 0.06 | 0.04 | 0.08 | 0.05 | -0.11 | -0.03 | -0.24 | -0.53 | 1 |  |  |  |  |  |  |  |  |  |  |  |
| **13** | 0.01 | 0.03 | -0.03 | -0.02 | 0.15 | 0.1 | 0.01 | -0.13 | 0.06 | -0.17 | -0.37 | -0.24 | 1 |  |  |  |  |  |  |  |  |  |  |
| **14** | -0.04 | 0.07 | 0.06 | 0.07 | 0.18 | 0.02 | -0.03 | 0.02 | -0.01 | 0 | -0.04 | 0.02 | 0.03 | 1 |  |  |  |  |  |  |  |  |  |
| **15** | -0.06 | -0.04 | 0 | 0 | 0.15 | -0.1 | 0.02 | 0.06 | 0.02 | -0.04 | -0.01 | 0.04 | 0 | 0.2 | 1 |  |  |  |  |  |  |  |  |
| **16** | -0.21 | -0.22 | -0.14 | -0.16 | 0.33 | 0.16 | -0.08 | -0.06 | -0.02 | 0.02 | 0.06 | -0.09 | 0 | 0.04 | 0.03 | 1 |  |  |  |  |  |  |  |
| **17** | 0.06 | 0.05 | 0.05 | 0.07 | -0.15 | -0.2 | -0.03 | 0.23 | -0.05 | 0.12 | 0.1 | -0.06 | -0.17 | 0.08 | 0.05 | 0 | 1 |  |  |  |  |  |  |
| **18** | -0.18 | -0.13 | -0.13 | -0.13 | 0.28 | -0.03 | 0.03 | -0.01 | 0.01 | -0.02 | 0.1 | -0.04 | -0.07 | 0.08 | 0.1 | 0.22 | 0.17 | 1 |  |  |  |  |  |
| **19** | -0.01 | -0.07 | 0.02 | 0.01 | 0.39 | 0.02 | 0.05 | -0.07 | 0.01 | 0.09 | -0.01 | -0.06 | 0 | 0.05 | -0.06 | 0.16 | 0.13 | -0.02 | 1 |  |  |  |  |
| **20** | -0.08 | 0.01 | 0.02 | 0.01 | -0.05 | -0.01 | -0.06 | 0.07 | 0 | 0.04 | -0.02 | 0 | -0.02 | 0.04 | 0.06 | 0.02 | 0.07 | 0.16 | -0.13 | 1 |  |  |  |
| **21** | 0.14 | 0.05 | 0.07 | 0.02 | 0 | -0.04 | 0.09 | -0.05 | -0.01 | 0.05 | 0 | -0.04 | 0.01 | -0.01 | -0.06 | -0.04 | 0 | -0.29 | 0.34 | -0.36 | 1 |  |  |
| **22** | -0.16 | -0.07 | -0.08 | -0.03 | -0.03 | 0.04 | 0.01 | -0.04 | -0.02 | -0.01 | 0.06 | -0.03 | -0.04 | -0.03 | -0.05 | 0.03 | 0.03 | 0.11 | -0.04 | -0.22 | -0.4 | 1 |  |
| **23** | 0.04 | 0 | -0.02 | 0 | 0.07 | 0.02 | -0.06 | 0.03 | 0.02 | -0.08 | -0.03 | 0.07 | 0.04 | 0 | 0.07 | 0 | -0.09 | 0.08 | -0.25 | -0.25 | -0.45 | -0.28 | 1 |
